# The functions and regulatory pathways of S100A8/A9 and its receptors in cancers

**DOI:** 10.3389/fphar.2023.1187741

**Published:** 2023-08-28

**Authors:** Huimin Zhou, Cong Zhao, Rongguang Shao, Yanni Xu, Wuli Zhao

**Affiliations:** ^1^ State Key Laboratory of Respiratory Health and Multimorbidity, Key Laboratory of Antibiotic Bioengineering, Ministry of Health, Laboratory of Oncology, Institute of Medicinal Biotechnology, Chinese Academy of Medical Sciences and Peking Union Medical College, Beijing, China; ^2^ NHC Key Laboratory of Biotechnology of Antibiotics, National Center for New Microbial Drug Screening, Institute of Medicinal Biotechnology, Chinese Academy of Medical Sciences and Peking Union Medical College, Beijing, China

**Keywords:** cancer, inflammation, RAGEs, S100A8/A9, TLR4

## Abstract

Inflammation primarily influences the initiation, progression, and deterioration of many human diseases, and immune cells are the principal forces that modulate the balance of inflammation by generating cytokines and chemokines to maintain physiological homeostasis or accelerate disease development. S100A8/A9, a heterodimer protein mainly generated by neutrophils, triggers many signal transduction pathways to mediate microtubule constitution and pathogen defense, as well as intricate procedures of cancer growth, metastasis, drug resistance, and prognosis. Its paired receptors, such as receptor for advanced glycation ends (RAGEs) and toll-like receptor 4 (TLR4), also have roles and effects within tumor cells, mainly involved with mitogen-activated protein kinases (MAPKs), NF-κB, phosphoinositide 3-kinase (PI3K)/Akt, mammalian target of rapamycin (mTOR) and protein kinase C (PKC) activation. In the clinical setting, S100A8/A9 and its receptors can be used complementarily as efficient biomarkers for cancer diagnosis and treatment. This review comprehensively summarizes the biological functions of S100A8/A9 and its various receptors in tumor cells, in order to provide new insights and strategies targeting S100A8/A9 to promote novel diagnostic and therapeutic methods in cancers.

## 1 Introduction

Some adverse conditions, such as dietary imbalance, tobacco smoking, pollutants, radiation, as well as bacterial and viral infections, can cause inflammation in multiorgan systems, eventually resulting in tumorigenesis and even necrosis. Inflammation is closely related to all stages of tumor development including initiation, growth, invasion, and survival, by mainly boosting genomic mutations, angiogenesis, and immunomodulation (Grivennikov et al., 2010; Hou et al., 2021). Various growth factors, cytokines, and chemokines are produced in the tumor microenvironment, which can bind to multitudinous cell surface receptors, like receptor for advanced glycation end products (RAGEs), vascular endothelial growth factor receptor (VEGFR), epidermal growth factor receptor (EGFR), and CXC chemokine receptor (CXCR), thus exerting diverse and profound effects via complex transduction ways including NF-κB, TGF-β, phosphoinositide 3-kinase (PI3K)/Akt, hypoxia-inducible factor (HIF) signaling, and reactive oxygen species (ROS) accumulation, in order to support the balance situation of tumor inflammation (Quail and Joyce, 2013; Libby and Kobold, 2019; Wattenberg and Beatty, 2020).

The S100 protein family is the principal participant in inflammation and includes 25 known members with a high degree of structural and sequence similarity. They act as intracellular and extracellular signal transduction factors bearing complicated and extensive physiological responsibilities, including but not limited to Ca^2+^ sensing, cell proliferation and differentiation, tumor development, cell cycle modulation, proinflammatory cytokine production, adipogenesis, and fatty acid transport (Bresnick et al., 2015; Gonzalez et al., 2020).

S100A8 and S100A9, also known as myeloid-related protein (MRP)8 or Calgranulin A and MRP14 or Calgranulin B, respectively, as well as the S100A8/A9 hetero-complex (Calprotectin), belong to the Ca^2+^-binding S100 protein family and are abundant in neutrophils and monocytes (Pruenster et al., 2016). As a significant inflammatory mediator, S100A8/A9 extensively influences granulocyte and monocyte migration, antibiosis, inflammatory propagation, macrophage activation, and cytostatic effect (Kerkhoff et al., 1998), playing prominent and complicated roles in myocardial infarction (Cai et al., 2020), cancer (Ghavami et al., 2009), coronavirus disease 2019 (Guo et al., 2021), tuberculosis (Scott et al., 2020), psoriasis (Matsunaga et al., 2021), and systemic lupus erythematosus (Lood et al., 2016). It is also a promising therapeutic target and clinical diagnostic biomarker for inflammatory bowel disease (Burri and Beglinger, 2014), rheumatoid arthritis (Hurnakova et al., 2015), type 2 diabetes (Ortega et al., 2012), acute Kawasaki disease (Hirono et al., 2006) and community-acquired pneumonia (Xie et al., 2023). Accordingly, we focused on the roles and mechanisms of S100A8/A9 in cancers to summarize the research advances, expecting to provide valid theoretical support for this study and future perspectives.

## 2 S100A8/A9 storage, structure, and effects

### 2.1 S100A8/A9 expression and release

S100A8/A9 is mainly stored in immune cells such as dendritic cells, neutrophils, monocytes, and macrophages, and is secreted to modulate inflammatory metabolic processes during infection, drug stress, heat, and other irritant conditions (Wang S. et al., 2018). Fibroblasts, keratinocytes, and vascular endothelial cells can also express S100A8/A9 upon activation (Schiopu and Cotoi, 2013). S100A8/A9 is predominantly localized in the cytoplasm and can translocate to the cytoskeleton and plasma membrane to increase intracellular calcium levels (Roth et al., 1993). S100A8/A9 constitutes approximately 40% cytosolic protein content in the main reserve source of neutrophils, and some S100A8 exists in granule membranes and euchromatin regions of the nucleus. S100A8/A9 isolated from neutrophils can further dose-dependently induce neutrophil activation and adhesion (Edgeworth et al., 1991; Sprenkeler et al., 2022).

At the same time, IL-17 (Bai et al., 2021), LPS (Fontaine et al., 2014), HIF-1 (Grebhardt et al., 2012), and tumor necrosis factor-α (TNF-α) (Nicaise et al., 2017) can also stimulate S100A8/A9 expression and release, while p38 MAPK signaling pathway (Mose et al., 2013), C/EBPα (Hayashi et al., 2007), protein kinase C (PKC) activation (Rammes et al., 1997), ROS production and K^+^ exchange (Tardif et al., 2015) are essential regulators for S100A8/A9 synthesis and secretion.

### 2.2 S100A8/A9 composition

Human S100A8 and S100A9 comprise 93 and 113 amino acids, respectively, with 10.8 and 13.2 kDa molecular weight, respectively. S100A8 and S100A9 monomers preferentially form stable heterodimers as their basic form, and two heterodimers can assemble into an (S100A8/A9)_2_ heterotetramer, which is indispensable for the basic biological functions of cells (Pruenster et al., 2016; Jukic et al., 2021).

**FIGURE 1:**
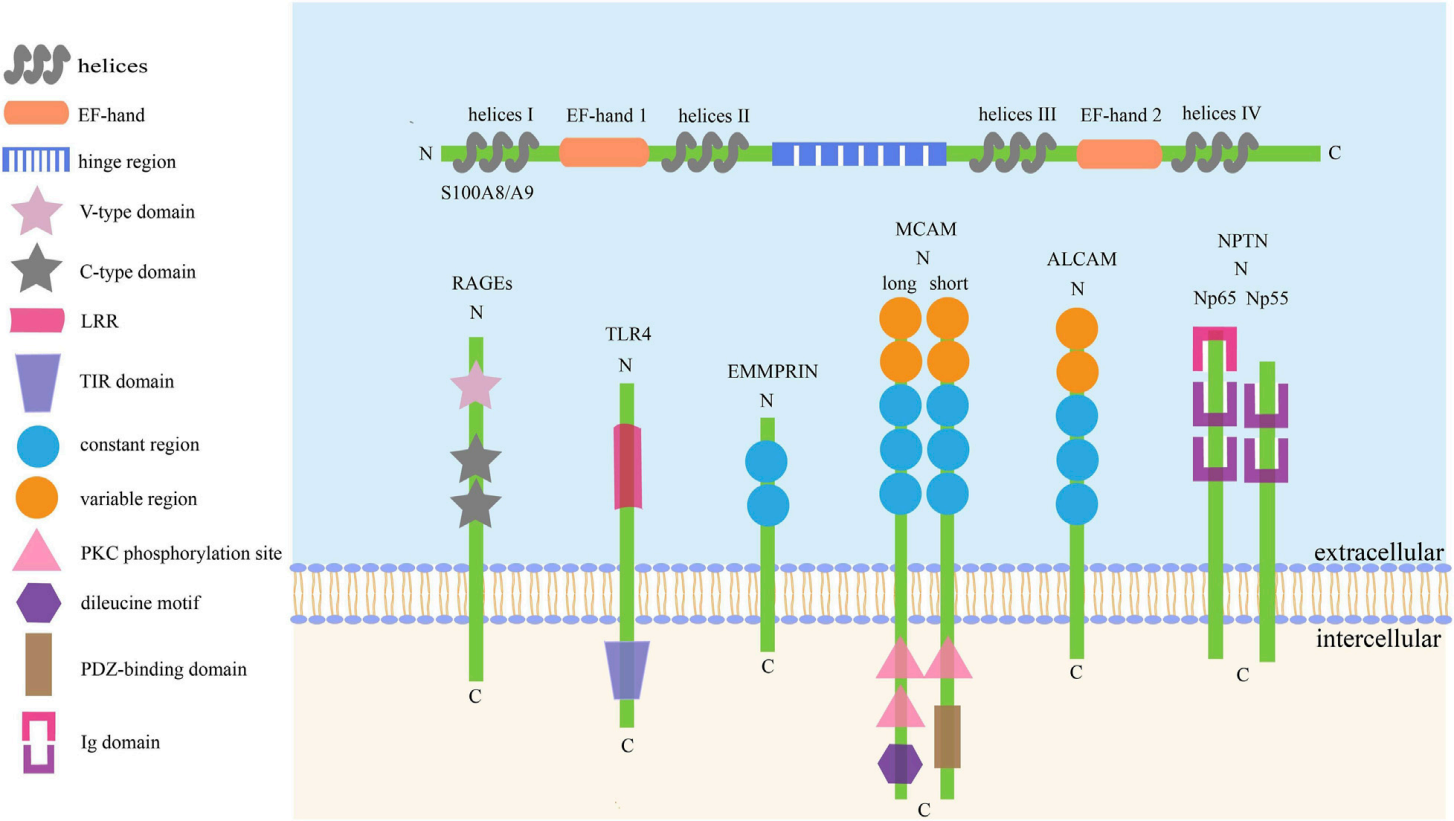
The basic structure of S100A8/A9 and its receptors. There are six kinds of receptors have been found to bind with S100A8/A9 to activate the downstream signaling axes, thus playing complex roles in cell activities. The common traits of these receptors are: I. They are collectively referred to as SSSRs. II. They are transmembrane proteins mainly consisting of three pivotal parts. III. Their activation can trigger similar signaling pathways, such as Akt, STAT3, ERK, and JNK.

Like other S100 proteins, S100A8/A9 is characterized by two EF-hand (such as helix–loop–helix)-type Ca^2+^-binding domains with different affinities to calcium, which are located at the N- (EF-hand 1, low affinity) and C-terminal (EF-hand 2, high affinity) halves, and connected with an intermediate region usually called the hinge region. Each Ca^2+^-binding site is flanked by two helices: EF-hand 1 is flanked by helices I and II, helices III and IV flank EF-hand 2, and the hinge region links helices II and III (Donato, 1999, 2001; Leukert et al., 2006; Korndörfer et al., 2007). The specific structure of S100A8/A9 is also described in Figure 1.

### 2.3 The fundamental functions of S100A8/A9

S100A8/A9 plays complex roles in chemotaxis (Ryckman et al., 2003), cell migration (Gross et al., 2014), inflammation (Tong et al., 2014), phagocytosis (Basu et al., 2018), and oxidative metabolism (Sroussi et al., 2010), involving sophisticated functions and mechanisms. First, being an important inflammatory factor, S100A8/A9 is involved in inflammation caused by injury, microorganisms, and stress. The released S100A8/A9 can promote IL-1β secretion in neutrophils in an autocrine or paracrine fashion to sustain the inflammatory response by stimulating myelopoiesis and other leukocyte production (Sreejit et al., 2020). The secreted S100A8 also stimulates myeloid cell chemotaxis by moving to inflammatory sites and sustaining leukocyte recruitment (Harrison et al., 1999).

Furthermore, calcium-induced S100A8/A9 co-localizes with microtubules in activated monocytes. It promotes microtubule formation to become more elongated and thread-like in resting phagocytes, and S100A8 is chiefly responsible for tubulin polymerization, whereas S100A9 plays important regulatory roles in this process. Together with p38 MAPK signaling pathway activation, calcium signaling-induced S100A8/A9 conformational changes can disrupt this stabilizing state of microtubules, thus facilitating the trans-endothelial migration of phagocytes (Vogl et al., 2004). Other studies have demonstrated that phosphorylation of S100A8/A9, superoxide production, and PKC modulation are essential for translocation from the cytosol to the membranes and cytoskeleton of S100A8/A9, which is of great importance for Ca^2+^-dependent reorganization of cytoskeletal filaments and inflammatory activation (Lemarchand et al., 1992; Goebeler et al., 1995; Guignard et al., 1996; van den Bos et al., 1996).

Moreover, S100A8/A9 heterodimers bind to arachidonic acid (AA) in a Ca^2+^-dependent manner and transfer it to nicotinamide adenine dinucleotide 3-phosphate (NADPH) oxidase to trigger enzymatic activity and stabilize the activated enzyme in neutrophils (Kerkhoff et al., 1999; Bai et al., 2022). Simultaneously, S100A8/A9 can also transfer AA to neighboring cells such as leukocytes, vascular endothelium, and smooth muscle cells at inflammatory loci to adjust the physiological state of the cells and produce inflammatory mediators to exacerbate inflammation (Kerkhoff et al., 2001; Kannan, 2003; Kerkhoff et al., 2005).

In addition to these functions, S100A8/A9 also participates in procoagulant platelet formation (Colicchia et al., 2022), intestinal microbiota development (Willers et al., 2020), autophagy-dependent ferroptosis (Tao et al., 2022), anemia, and bone marrow failure (Giudice et al., 2019), most of which are closely related to immunity and inflammation, indicating that S100A8/A9 may serve as a biomarker and therapeutic target in this field.

## 3 S100A8/A9 has complicated influence on cancer cells

S100 proteins widely participate in cellular proliferation, metabolism, inflammation, and many other diverse physiological activities by EF-hand binding to calcium, zinc, or copper to change the spatial conformation and interact with other target proteins to activate downstream signals (Oesterle and Bowman, 2015). In cancer cells, S100A8/A9 is the main modulator of apoptosis (Qin et al., 2010), migration, invasion (Yin et al., 2013), brain metastasis (Monteiro et al., 2022), drug resistance (Zavorka Thomas et al., 2021) and many other processes, acting as a dual and diverse sensor in different steps of tumor progression, some of which are described below.

### 3.1 S100A8/A9 is overexpressed in carcinoma and has negative outcomes in patients

According to patient tissue microarrays and immunohistochemical detection, both S100A8 mRNA and protein levels are highly elevated in many cancers, including anaplastic thyroid carcinoma (Reeb et al., 2015), breast cancer (Wang D. et al., 2018), lung cancer (Sumardika et al., 2019), acute myeloid leukemia (Mondet et al., 2021), and pancreatic cancer (Basso et al., 2014), and associated with less survival, poor outcome, and high probability of relapse; thus, S100A8 could act as a potential biomarker for different cancers. Simultaneously, S100A9 expression is also elevated in the tissues or sera of colorectal carcinoma (Huang et al., 2019), hepatocellular carcinoma (Zhong et al., 2022), gastric cancer (Fan et al., 2012), nasopharyngeal carcinoma (Hu et al., 2021), and renal cell carcinoma (Zhang et al., 2015), consistent with low disease-free, overall, and recurrence-free survival periods, which can also be used as a powerful diagnostic indicator (Koh et al., 2019; Koh et al., 2021). The DNA methylation level of S100A8 decreases in hepatocellular carcinoma tissues compared to that in adjacent normal tissues, which is closely related to reduced overall and progression-free survival periods, indicating that S100A8 DNA methylation could also be a useful diagnostic and prognostic marker (Liu K. et al., 2016). Thus, incorporating S100A8/A9 as a supplementary biomarker will aid the early detection and timely treatment of cancers.

### 3.2 S100A8/A9 plays important roles in the proliferation of cancer cells

Since proliferation is one of the most important tumor characteristics, many studies have focused on the function of S100A8/A9 in it. S100A8/A9 overexpression notably improves the viability of nasopharyngeal carcinoma cells, and S100A8/A9 knockdown represses proliferation and colony formation by inhibiting the PI3K/Akt pathway (Wen et al., 2021). Exogenous S100A8/A9 can bind to RAGEs to induce squamous cell carcinoma proliferation, and direct interruption by a specific RAGE-blocking antibody can reduce cellular proliferation through MAPK phosphorylation alteration (Iotzova-Weiss et al., 2015). Moreover, S100A8/A9 induces the apoptosis of chronic eosinophilic leukemia cells by suppressing the downstream signal of FIP1L1-PDGFRα through decreasing its mRNA and proteins expression, as well as eliciting the activity of mitochondria-associated proteins such as caspase 3/9, AIF, and Bax (Lee et al., 2020). In an orthotopic lung cancer mouse model, intranasal S100A8 delivery effectively delayed tumor growth and prolonged survival by inducing the antioxidant activity of many key genes and activating immune cells to positively modify the immune microenvironment (Wong et al., 2022). The distinct roles of S100A8/A9 in tumor proliferation might be consistent with its concentration and the tumor cell environment. If S100A8/A9 cooperates with other immune factors to activate suppressive pathways in tumor cells, it would lead to cell death. Moreover, S100A8/A9 concentration lower or higher than a particular concentration might induce the proliferation or apoptosis of tumor cells, respectively. Therefore, the specific concentration and pathways involved must be further explored.

### 3.3 S100A8/A9 has a dual effect on the migration and invasion of cancer cells

Metastasis is a basic property of tumor cells. In colorectal cancer (CRC) cells, signaling factors TGF-β can induce S100A8 expression, thus promoting the movement of CRC cells. However, cell migration and invasion significantly decrease after S100A8 knockdown, which is not restored by TGF-β (Li et al., 2021). The addition of recombinant human S100A8/A9 protein promotes esophageal squamous cell carcinoma cell migration and invasion via the Akt and p38 MAPK signaling pathways, whereas S100A8/A9 silencing suppresses carcinoma cell migration and invasion, as well as Akt and p38 MAPK activation (Tanigawa et al., 2022). Simultaneously, the exogenous S100A8/A9 complex promotes the migration of triple-negative breast cancer cells by activating the RAGE–FAK–Hippo/Yes kinase-associated protein (YAP) transduction system (Rigiracciolo et al., 2022). In animal models, compared to controls, anaplastic thyroid carcinoma cells with S100A8 knockdown showed less lung metastasis and tumor load by tail vein injection, whereas S100A9 knockdown did not affect tumorigenesis and metastasis (Reeb et al., 2015). In addition, many studies have shown that Toll-like receptor 4 (TLR4), NF-κB, matrix metalloprotease-2 (MMP-2), and other proteins also participate in S100A8/A9-mediated cancer cell migration and invasion through different transduction pathways (Kwon et al., 2013; Silva et al., 2014; Herik Rodrigo et al., 2022). Moreover, intracellular S100A8 can suppress invasion and epithelial–mesenchymal transition (EMT) in breast cancer cells (Cormier et al., 2014). This might be related to the source of S100A8/A9 as extracellular could bind to specific receptors to activate certain downstream pathways, whereas intracellular S100A8/A9 might be regulated in completely different ways. At the same time, the inflammatory microenvironment also participates in this process, where cytokines could trigger crossing pathways to push forward to entirely different cell fates, along with positively or negatively modulating S100A8/A9 effects, and the detailed role of S100A8/A9 for tumor metastasis need to be explored separately.

### 3.4 S100A8/A9 contributes to angiogenesis in cancer cells

Some studies have shown that low S100A8/A9 concentrations can not only stimulate the proliferation and migration of vascular endothelial cells *in vitro*, but also promote tumor cell vascularization to benefit their growth (Li et al., 2012; Zhong et al., 2020). Studies have shown that myeloid cell-derived S100A9 can increase blood supply and promote angiogenesis by inducing IL6 and IL10 to create a survival niche for subsequent tumor progression in multiple myeloma (De Veirman et al., 2017). Meantime, S100A9 activates angiogenesis via an autocrine effect on tumor cells and a paracrine effect on stromal cells in oral cancer, similarly accompanied by IL6 production (Fang et al., 2015). In addition, studies using a mouse lung cancer model have found that the S100A8/S100A9–EMMPRIN–Vegfa axis promotes tumor angiogenesis and progression, which is associated with the prognosis of lung cancer patients (Nguyen et al., 2021). Although the promotional effect of S100A8/A9 on angiogenesis has been confirmed by many studies, whether S100A8/A9 could induce nutritional deprivation under some unique conditions and be targeted to prevent angiogenesis remains unclear, and its function in tumor angiogenesis needs to be further investigated.

### 3.5 S100A8/A9 promotes immunosuppression of cancer cells

In addition to the complex functions mentioned above, S100A8/A9 plays a major role in tumor immunology and metabolic activity (Wu et al., 2022). Programmed death 1 (PD-1) is an important co-inhibitory signal, and tumor or immune cells express PD-1 ligands (PD-L1) to evade antitumor immune responses (Lei et al., 2020). Studies have shown that S100A8 expression is positively correlated with PD-L1 levels in tumor samples and can induce PD-L1 in macrophages by activating PI3K/Akt, MAPKs, NF-κB, and STAT3 to increase PD-L1 transcription. Furthermore, S100A8-treated macrophages cannot promote T cell proliferation *in vitro* and can suppress the cytotoxic function of tumor-primed T cells *in vivo* through PD-L1 (Li et al., 2020). Breast cancer-associated gene 1 (BRCA1) is a familiar tumor suppressor gene, and BRCA1 deficiency can contribute to various breast cancers. Moreover, BRCA1 mutations can activate the S100A9–CXCL12–pSTAT3 axis to induce myeloid-derived suppressor cells (MDSCs) accumulation, further establishing an immunosuppressive environment for tumorigenesis, and blockade of S100A9–CXCL10 could diminish immunosuppressive niches and inhibit cancer initiation and growth in the mouse model (Li et al., 2022). In addition, S100A9–RAGEs–p38 MAPK and S100A9–TLR4–NF-κB also participate in MDSCs activation and chemotaxis *in vitro*, which are closely related to immunosuppression and neoplastic progression (Huang et al., 2019). In recent years, tumor immunotherapy has become an emerging research hotspot. Thus, it is inspiring to find that S100A8/A9 takes part in immunomodulatory processes and particularly has a positive correlation with PD-L1, which might help us find new molecular mechanisms to detect immune evasion; however, further elucidation is needed.

### 3.6 S100A8/A9 increases the chemoresistance of cancer cells

Drug resistance is one of the important problems in cancer therapy. Previous studies have shown that recombinant human S100A8/A9 can reduce the cytotoxicity of doxorubicin/cyclophosphamide in breast cancer cells (Hsu et al., 2015). Moreover, S100A9 overexpression can lead to resistance to cisplatin-induced apoptosis in bladder cancer, whereas S100A9 inhibition can enhance tumor cell sensitization to cisplatin-induced apoptosis (Kim et al., 2014). Interleukin 1 receptor-associated kinase 1 (IRAK1) is a serine/threonine-protein kinase that mediates IL1–TLR–NF-κB signaling pathway; and IRAK1–S100A9 axis plays a critical role in the paclitaxel resistance in nasopharyngeal carcinoma, while S100A9 ablation could increase the sensitivity of cells to paclitaxel, and the addition of recombinant S100A9 could rescue the resistance to paclitaxel after S100A9 knockdown (Liu et al., 2021). IL6 is an essential driver of S100A8/A9 expression in acute myeloid leukemia cells via JAK/STAT3 signaling; and acute myeloid leukemia populations with elevated S100A8/A9 levels are highly resistant to doxorubicin, which can be efficiently reversed by blocking JAK/STAT3 signaling (Böttcher et al., 2022).

Autophagy is a catabolic process involving the formation of autophagosomes that engulf proteins or organelles and the translocation of autophagosomes to lysosomes for degradation, which also causes chemotherapeutic resistance (Levy et al., 2017). Previous studies have reported that S100A8 triggers autophagy by promoting BECN1–PI3KC3 complex expression and BECN1–BCL2 complex formation inhibition to maintain the strong resistance of B-cell lymphoma cells to adriamycin and vincristine (Zhang et al., 2021). Simultaneously, S100A8 knockdown decreases LC3 conversion, p62 degradation, and autophagosome maturation with ROS participation in leukemia cells, thus inhibiting the initiation of autophagy to increase chemotherapy sensitivity (Yang et al., 2012). In view of the facilitation of drug resistance by S100A8/A9, S100A8/A9 antagonists might be developed to increase the vulnerability of tumor cells to chemotherapy, and the combined use of autophagy inhibitors might have excellent therapeutic effects. All concepts must be further tested and verified.

## 4 Main S100A8/A9-binding receptors and molecular pathways in cancer cells

**FIGURE 2:**
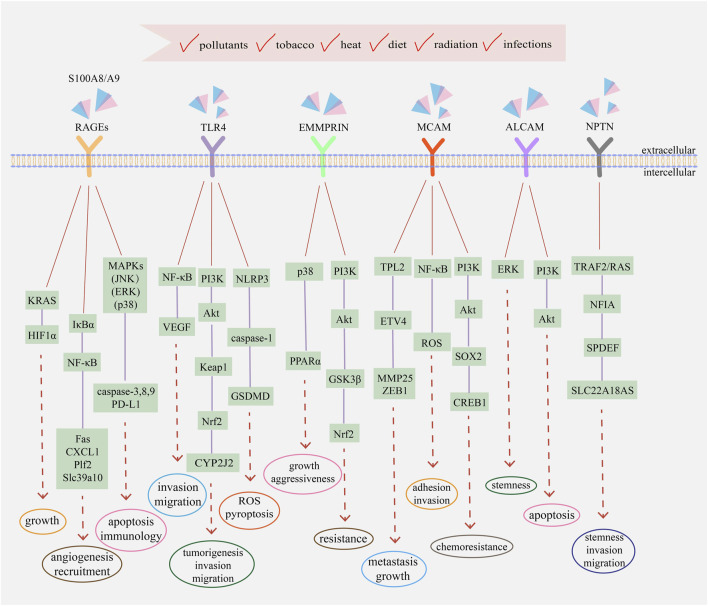
The main transduction pathways involving with S100A8/A9 and its receptors in cancers. Extracellular S100A8/A9 can bind to different receptors and activate signaling pathways to regulate the progression and development of tumor cells, including proliferation, metastasis, drug resistance, immune evasion, and angiogenesis. Intracellular S100A8/A9 also participates in the activation of MAPKs, NF-κB, PI3K/Akt, and other transduction pathways. Simultaneously, intracellular and extracellular cytokines and kinases assist the functions of S100A8/A9 and jointly regulate the eventual fate of tumor cells, depending on the comprehensive effects of physiological status, environmental conditions, and external incentives.

Since S100A8/A9 functions like soil signal and the paired receptors act like soil sensor, S100A8/A9 receptors, including RAGEs, TLR4, extracellular matrix metalloproteinase inducer (EMMPRIN), melanoma cell adhesion molecule (MCAM), activated leukocyte cell adhesion molecule (ALCAM), and neuroplastin (NPTN), have a common name ‘S100 soil sensor receptors (SSSRs)’ (Kinoshita et al., 2019a; Tomonobu et al., 2020). These receptors are always used as biomarkers for the diagnosis and prediction of idiopathic pulmonary fibrosis (Machahua et al., 2018), proliferative diabetic retinopathy (Abu El-Asrar et al., 2021), lupus nephritis (Lei et al., 2022), obstructive sleep apnea (Sun et al., 2020), and subarachnoid hemorrhage (Heinz and Schneider, 2022). Simultaneously, the S100A8/A9–SSSRs axis participates in many aspects of tumor initiation and development, usually involving MAPKs, NF-κB, PI3K/Akt, and mTOR along with the assistance of VEGF, YAP, Nrf2, and HIF-1 (Jiang et al., 2012; Tang et al., 2015). The structure and regulatory pathways of SSSRs are introduced below, which are also summarized in Figures 1, 2 respectively.

### 4.1 RAGEs

RAGEs, mainly located on the surfaces of macrophages, endothelial cells, dendritic cells, and tumor cells, act as pattern-recognition receptors and belong to the immunoglobulin (Ig) superfamily (Ott et al., 2014). They distinguish and bind to many different ligands, including high-mobility group box-1 (HMGB1), amyloid-peptide, S100 proteins, and advanced glycation end products (AGEs), triggering numerous downstream signal transductions (El-Far et al., 2020). The full-length RAGE is probably composed of a large extracellular domain, a single transmembrane-spanning helix, and a short cytoplasmic region essential for signal transduction (Matsumoto et al., 2008). The extracellular region consists of one N-terminal variable (V-type) domain and two constant (C-type) domains, and the ligand-binding site is located on the V-type domain and predominately folded into a two β-sheet and a conserved pair of cysteines with a disulfide bond (Dattilo et al., 2007).

S100 proteins can bind to RAGEs to modulate tumor cell growth, survival, and invasion, accompanied by the activation of various signal transduction pathways, such as JNK, P38 MAPK, ERK, NF-κB, and PI3K/Akt (Ahmad et al., 2018). Previous studies have reported that the P38 MAPK and JNK pathways can induce cell apoptosis through caspase activation and their inhibitors can alter caspase-3, -8, and -9 expression (Liu T. et al., 2016). Meanwhile, exogenous S100A8/A9-induced ERK1/2 phosphorylation can promote the proliferation and invasion of tumor cells (Iotzova-Weiss et al., 2015). IκBα phosphorylation and NF-κB activation due to low S100A8/A9 concentrations usually play indispensable roles in the development of inflammation-based tumors; namely, nuclear translocation of NF-κB could promote the expression of many pro-tumorigenic genes, like apoptotic gene Fas, chemokines CXCL1, growth factor Plf2, and zinc transporter Slc39a10, actively participating in angiogenesis and recruitment (Ichikawa et al., 2011). Under hypoxic conditions, oxidative stress, and inflammation, RAGE activation sustains the KRAS signaling pathway to facilitate the stabilization and transcriptional activity of HIF1α, resulting in pancreatic tumor growth. RAGE knockdown and KRAS signaling inhibition can promote HIF1α degradation and increase hypoxia-induced tumor cell death (Kang et al., 2014). Since RAGEs are the most common S100A8/A9 receptors, when focusing on traditional targets to screen new drugs, we need to focus on innovative signaling pathways to find novel methods to meet emerging clinical needs.

### 4.2 TLR4

To date, 10 different and functional TLRs have been found in humans. Structurally, TLRs are transmembrane domain proteins consisting of three core parts: an ectodomain of leucine-rich region (LRR), transmembrane, and a cytoplasmic domain of Toll/interleukin-1 receptor (TIR) domain that recruits MyD88 and TRAM adapter to activate downstream NF-κB, AP-1, and MAPKs signaling axes (Mishra and Pathak, 2019; Kashani et al., 2021).

S100A8/A9 can bind to TLR4 and induce many various signal transduction pathways, including ERK, p38 MAPK, PKC, NF-κB, TNF-α, and IRAK1 (Ehrchen et al., 2009). S100A8 overexpression in cholangiocarcinoma could facilitate *in vitro* and *in vivo* cell invasion and migration by activating the NF-κB pathway as well as increasing VEGF expression, while S100A8 knockdown or TLR4 and NF-κB inhibition could significantly inhibit cell migration and metastasis (Pan et al., 2020). Meantime, after TLR4/Akt activation, the Keap1/Nrf2 cascade can promote tumorigenesis, invasion, and migration of CRC cells by upregulating cytochrome P450 monooxygenase CYP2J2 expression (Kong et al., 2021). Moreover, TLR4 activation can trigger pyroptosis in non-small cell lung cancer *in vivo* and *in vitro* by promoting mitochondrial ROS generation and Ca^2+^ aggregation in an NLRP3/caspase-1/GSDMD-dependent manner (Yuan et al., 2021). The Apc ^Min/+^ mouse model is a well-utilized intestinal cancer model. TLR4 can promote intestinal tumorigenesis by activating cytokine–cytokine receptor interactions, while TLR4 knockdown can significantly decrease the expression of S100A8/A9 and other factors to inhibit the formation of intestinal adenomas and prolong the life of Apc ^Min/+^ mice (Shi et al., 2020). Although many studies have focused on the relationship and effects of TLR4 and S100A8/A9 in cancer, the mechanisms that could be developed as drug targets are not well-reported, and research in this area should be constantly strengthened.

### 4.3 EMMPRIN

EMMPRIN, also called CD147 or basigin, serves as a receptor for calprotectin in tumor cells and has a higher affinity for S100A9 than that for S100A8 (Hibino et al., 2013). EMMPRIN activation will mainly initiate PI3K, MAPKs, and NF-κB pathways, promoting the growth, angiogenesis, dissemination, and inflammation of tumor cells (Xin et al., 2016; Dana et al., 2021). It comprises 269 amino acid residues and can be divided into three core parts: an extracellular N-terminal domain containing two C2-type Ig regions responsible for activating matrix metalloproteinases and becoming glycosylated, a hydrophobic transmembrane domain acting as a signal peptide, and a C-terminal intracellular cytoplasmic domain responsible for association with integrins (Bai et al., 2014; Xiong et al., 2014).

EMMPRIN is overexpressed in penile carcinoma with a remarkable influx of S100A8^+^S100A9^+^ immune cells, resulting in intracellular signaling that enhances the expression of pro-tumorigenic genes (Mohr et al., 2022). Simultaneously, EMMPRIN expression on CD8^+^ tumor-infiltrating lymphocytes is significantly elevated, which cooperates with other receptors to negatively regulate antitumor immune responses and facilitate tumor-immune escape, whereas EMMPRIN deficiency can inhibit tumor growth and enhance the effector function of T cells (Chen Y. et al., 2021). Protein methylation is an important post-translational modification; methylated EMMPRIN is overexpressed in non-small cell lung cancer and enhances glycolysis and lactate export to promote cancer progression *in vivo* and decrease the overall survival of patients (Wang et al., 2021). EMMPRIN also influences the drug resistance of tumor cells. Temozolomide is a common chemotherapeutic agent for glioma treatment. Overexpressed EMMPRIN can inhibit Akt/GSK3β-dependent Nrf2 degradation to promote Nrf2-mediated antioxidant gene expression, which inhibits temozolomide-triggered ROS production and cell death, ultimately resulting in the resistance of glioma cells to temozolomide (Bu et al., 2021). Reprogramming of lipid metabolism is also a representative characteristic of tumor cells. EMMPRIN could promote *de novo* lipogenesis via the upregulation of lipogenic enzymes by activating the Akt/mTOR signaling pathway as well as suppress fatty acid oxidation via downregulating the expression of fatty acid oxidative enzymes and PPARα by the p38 MAPK signaling pathway, thus increasing the growth and aggressiveness of hepatocellular carcinoma cells (Li et al., 2015). Unlike RAGEs and TLR4, EMMPRIN plays an important role in tumor immunity and metabolism, in which field can be used as a target to design clinical drugs. Combined with drug sensitizers such as autophagy inhibitors, it might have significant antitumor effects.

### 4.4 MCAM

MCAM, originally identified as CD146, is a cell adhesion molecule involved in various tumor physiological activities including angiogenesis, immune response, and migration (Wang and Yan, 2013; Wang et al., 2020). Structurally, CD146 is a 113 kDa transmembrane glycoprotein with three main parts: an N-terminal extracellular domain, a transmembrane domain, and a long (lgCD146) and short (shCD146) C-terminal cytoplasmic domain. The extracellular portion contains five Ig-like domains, consisting of two variable regions (V) and three constant regions (C2) (V–V–C2–C2–C2), including eight potential N-glycosylation sites. lgCD146 and shCD146 are generated by alternative splicing and have different functions: the longer one contains two phosphorylation sites for PKC and a dileucine motif for CD146 internalization and trafficking, and the shorter one has one PKC phosphorylation site and one PDZ-binding domain for cell signaling (Lei et al., 2015; Leroyer et al., 2019; Rapanotti et al., 2021).

S100A8/A9 can bind to MCAM, which is overexpressed in triple-negative malignant breast cancer, promoting cell EMT phenotype and mortality by activating the S100A8/A9–MCAM–ETV4–ZEB1 axis, along with MCAM knockdown leading to a reduced response to S100A8/A9 and downregulation of migration ability (Chen et al., 2019a). In melanoma cells, activation of the S100A8/A9–MCAM–TPL2–ETV4–MMP25 axis promotes growth and lung metastasis *in vivo* (Chen et al., 2019b). Moreover, NF-κB activation and ROS formation usually play pivotal roles in MCAM-mediated adhesion and invasion capacity in cancer progression (Ruma et al., 2016). Glucosaminyl (N-acetyl) transferase 3 (GCNT3) is a glycosyltransferase that is overexpressed in various cancer cells. It promotes the maintenance of MCAM protein at high levels and elevates the stability of MCAM by glycosylation, thus maintaining the responsiveness of MCAM to S100A8/A9 to increase melanoma cellular migration and invasion (Sumardika et al., 2018). MCAM also mediates chemoresistance in small-cell lung cancer and is upregulated in chemo-resistant cells. MCAM knockdown can increase chemosensitivity, induce cell cycle arrest, and promote apoptosis by increasing p-BAD and caspase-3 expression. PI3K/Akt/SOX2/CREB1 signaling and glycolytic shifts also participate in MCAM-guided chemoresistance in cancer cells (Tripathi et al., 2017). MCAM can also promote angiogenesis to supply nutrients for tumorigenesis, and MCAM inhibition can increase the penetration of chemotherapeutic drugs, indicating that MCAM may be used as a new therapeutic target (Lei et al., 2021). Owing to the widespread involvement of MCAM in various aspects of tumor development, drugs targeting MCAM are promising and meaningful, as they may act from multiple perspectives rather than on a specific aspect of tumors, potentially having strong antitumor effects.

### 4.5 ALCAM

ALCAM also called CD166, is a pivotal cell adhesion protein mediating migration, growth, and adhesiveness in cancer cells involving NF-κB, β-catenin, and Twist signaling, as well as has complicated clinical prognostic significance in different cancers (Yang et al., 2021). ALCAM is a transmembrane molecule divided into three parts: a large N-terminal extracellular domain (ED), a transmembrane region, and a short cytoplasmic domain. The ED has five Ig-like domains (V–V–C2–C2–C2) consisting of three membrane-proximal constant regions (C2) and two membrane-distal variable regions (V), containing 10 potential N-glycosylation sites. The Ig-like domains are mainly responsible for regulating the affinity of homophilic ALCAM–ALCAM and heterophilic ALCAM–CD6 interactions. Cell–cell adhesion is mediated by trans-ALCAM–ALCAM interactions, which touch the amino-terminal ligand-binding modules (V-domains) (von Lersner et al., 2019; Darvishi et al., 2020; Ferragut et al., 2021).

In non-small-cell lung cancer, ALCAM expression is significantly elevated and closely linked to short overall survival and poor prognosis. ALCAM contributes to brain metastasis by interacting with the brain vascular endothelium (Münsterberg et al., 2020). Simultaneously, ALCAM acts as a critical anti-apoptotic regulator by activating PI3K/Akt signaling, upregulating YAP protein expression, and downregulating Bcl-2 expression in liver cancer cells (Ma et al., 2014). ALCAM can also enhance the cancer stem cell-like (CSC) phenotype of nasopharyngeal carcinoma by activating the EGFR/ERK1/2 signaling pathway, and ALCAM downregulation decreases tumor sphere formation, thus inhibiting CSC-like characteristics and weakening tumorigenesis capacity *in vivo* (Chen X. et al., 2021). ALCAM is also associated with tamoxifen resistance in breast cancer cells, and ALCAM knockdown substantially enhances tamoxifen-induced cancer cell viability inhibition and apoptosis (Chen et al., 2017). Moreover, ALCAM mediates the resistance of prostate cancer to enzalutamide (ENZA), a second-generation androgen receptor inhibitor frequently used for treating metastatic castration-resistant prostate cancer and ALCAM gene silencing in drug-resistant cells could enhance ENZA sensitivity (Csizmarik et al., 2022). Drug research targeting ALCAM is relatively common, with most achieving ideal results; however, it is limited to laboratory studies and cannot be applied in clinical practice. Currently, there is an urgent need to use drugs with good therapeutic effects in clinics.

### 4.6 NPTN

NPTN or Np is a transmembrane glycoprotein belonging to the Ig superfamily of cell adhesion molecules and has three domains: extracellular Ig domains, transmembrane domains, and short cytoplasmic domains. NPTN has two splicing variants, NPTNα and NPTNβ, also named Np55 and Np65 owing to their apparent molecular weights. In their extracellular structure, NPTNα has two Ig domains, while NPTNβ has three, and the extra domain is useful to bind with S100A8 and Np65 homophilic interactions. Notably, S100A8 only binds to NPTNβ, while S100A9 can bind to NPTNα (Owczarek and Berezin, 2012; Sakaguchi et al., 2016; Ilic et al., 2021).

In lung cancer cells, S100A8/A9 can bind to overexpressed NPTNβ, activating transcription factors NFIA by TRAF2 and RAS signaling, and ultimately promotes cellular spheroidal growth, migration, and invasion through downstream molecules SAM pointed-domain containing E26 transformation-specific transcription factor (SPDEF) (Sumardika et al., 2019). SPDEF plays a vital role in NPTNβ functions and the NPTNβ–SPDEF-induced solute carrier family 22 member 18 antisense (SLC22A18AS) is essential for EMT and cellular motility in lung cancer, with a negative correlation with overall survival of patients (Bajkowska et al., 2020). Moreover, in breast cancer cells, overexpressed-NPTN contributes to tumor growth and angiogenesis *in vivo*, which is related to the increased VEGF production in microenvironmental hypoxia (Rodriguez-Pinto et al., 2009). Compared with that of other receptors, the functions and related mechanisms of NPTN in tumor cells are relatively less known, and further studies should aim to clarify the involvement of NPTN in the growth, migration, and angiogenesis of different cancers.

## 5 Potential S100A8/A9-targeting strategies to treat cancers

Since S100A8/A9 is a prominent mediator of cancers, targeting S100A8/A9 to explore novel strategies in tumor treatment is promising. S100A8/A9 can be used as an effective tool to detect early-phase inflammation and tissue damage by anticancer agents (Morikawa-Ichinose et al., 2022). Using specific antibodies, changes of S100A8/A9 in tumor-bearing mice can be monitored to evaluate the establishment of an immunosuppressive state and a metastasis-permissive microenvironment (Eisenblaetter et al., 2017). In addition, comparing the expression of S100A8 in tumor cells and immune cells at different stages can help determine the clinicopathological features and clinical outcomes of patients (Woo et al., 2021).

**FIGURE 3:**
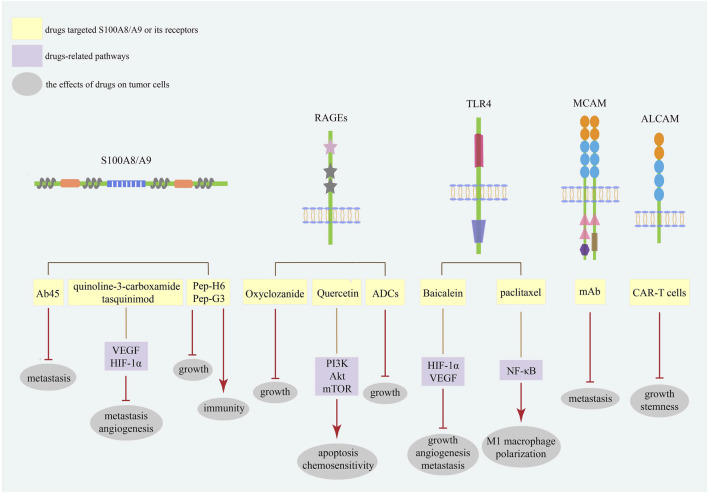
The strategies targeting S100A8/A9 and its receptors in cancers. The strategies of S100A8/A9 and its receptors in cancer treatment mainly focus on neutralizing S100A8/A9 and blocking its binding with receptors. Current studies have focused on the most common receptors RAGEs and TLR4. These targets are promising diagnostic and prognostic biomarkers for various cancer types.

Considering the increasing plasma levels of S100A8/A9 in malignancies, blocking the interaction between S100A8/A9 and its receptors seems likely to be an efficacious therapeutic method (Figure 3). Oxyclozanide, a chemical probe and inhibitor of S100A9 and RAGEs, can inhibit tumor growth *in vivo* (Björk et al., 2013). Ab45, an S100A8/A9 heterodimer-specific neutralizing antibody, efficiently blocks melanoma lung metastasis (Kinoshita et al., 2019b). Meanwhile, quinoline-3-carboxamide tasquinimod, a strong S100A9 binder, can inhibit the establishment of primary tumors and hinder the formation of distant metastatic niches in prostate cancer, even breaking the angiogenic balance by downregulating VEGF and HIF-1α (Jennbacken et al., 2012). The therapeutic peptides Pep-H6 and Pep-G3, which recognize extracellular S100A8/A9 on MDSCs surface, could completely deplete blood and intra-tumoral MDSCs, thus enhancing the antitumor immunity of tumor-bearing mice and inhibiting tumor growth *in vivo*, indicating an emerging treatment direction for S100A8/A9 together with immune evasion (Qin et al., 2014).

Meantime, different S100A8/A9 receptors may serve as potential therapeutic targets (Figure 3). Baicalein directly binding to TLR4 could inhibit HIF-1α/VEGF signaling pathway to reduce the growth, angiogenesis, and metastasis of CRC cells (Chen M. et al., 2021). Meanwhile, paclitaxel can promote tumor regression via TLR4/NF-κB signaling in melanoma by inducing M1 macrophage polarization (Wanderley et al., 2018). Quercetin induces autophagy, apoptosis, and chemosensitivity by RAGE/PI3K/Akt/mTOR pathways in pancreatic cancer cells (Lan et al., 2019). Antibody–drug conjugates (ADCs) against RAGEs kill endometrial tumor cells both *in vitro* and *in vivo* (Healey et al., 2019). Since MCAM has a positive effect on cancer metastasis, studies have demonstrated that MCAM knockdown can reduce lytic bone metastasis in preclinical mouse models of prostate cancer, and MCAM-targeted mAbs can effectively postpone intraosseous metastatic lesions (Zoni et al., 2019). Recently, chimeric antigen receptor (CAR)-T cells with remarkable clinical responses and high specificity have become a new research hotspot for cancer treatment (Sterner and Sterner, 2021). In osteosarcoma cells, modified ALCAM CAR-T cells exhibit specific and potent cytotoxicity, induce regression of orthotopic osteosarcoma *in vivo*, and have no cytotoxic activity against healthy tissues (Wang et al., 2019). Moreover, ALCAM-specific CAR-T cells can efficiently lyse CRC cells and show powerful cytotoxicity against CRC stem cells, demonstrating a feasible approach for reconstructing CAR-T cells with S100A8/A9 and its receptors for cancer therapy (He et al., 2023).

Despite the endless emergence of drugs targeting S100A8/A9 and its receptors, which substantially inhibit different characteristics of tumor cells, they are only at the level of theoretical research, regrettably, there are no drugs that can be applied in practical life. The main problem is that the functional explanation of S100A8/A9 and its receptors is incomplete, very few studies have focused on the relevant mechanisms to support its clinical application. Thus, there is an urgent need to deeper study S100A8/A9 and its receptors to identify new strategies.

## 6 Summary

S100 proteins are involved in various human physiological activities, such as energy metabolism, cellular composition, metal ion transportation, and signal transmission. S100A8/A9 is an inflammatory mediator that broadly participates in many diseases, and can act as a useful biomarker for diagnosis and prediction. S100A8/A9 can regulate the initiation, proliferation, invasion, metastasis, angiogenesis, chemoresistance, and apoptosis of tumor cells by binding with its receptors scattered in tumor cell surface, including RAGEs, TLR4, EMMPRIN, MCAM, ALCAM, and NPTN, mostly by activating the JNK, ERK, P38 MAPK, PI3K/Akt, NF-κB, TGFβ, and STAT3 signal transduction pathways; thus, they can be potential targets for cancer treatments.

Currently, some tactics aimed at S100A8/A9 and its receptors in cancer treatments have been studied, such as chemical drugs, specific antibodies, antagonists, and peptides. However, further studies should aim to clarify the relevance and function of S100A8/A9 and its receptors in cancers or other serious disorders, such as the balance of promotion and inhibition to cancers, new effects on oncogenesis, crosstalk of different receptors and concrete mechanism, correlation with tumor microenvironment, synergistic or antagonism cooperation with inflammatory factors, as well as discovery of feasible drug targets, which could open a new door leading to a brand new era when cancer may be disappearing.

## Figures and Tables

**Table:** 

**AA**	Arachidonic acid
**ADCs**	Antibody-drug conjugates
**AGEs**	Advanced glycation end-products
**AIF**	Apoptosis-inducing factor
**ALCAM**	Activated leukocyte cell adhesion molecule
**BRCA1**	Breast cancer-associated gene 1
**CAR**	Chimeric antigen receptor
**CRC**	Colorectal cancer
**CREB**	CAMP-response-element-binding protein
**CSC**	Cancer stem cell
**CXCL12**	CXC chemokine ligand 12
**CXCR**	CXC chemokine receptor
**ED**	Extracellular domain
**EGFR**	Epidermal growth factor receptor
**EMT**	Epithelial-mesenchymal transition
**EMMPRIN**	Extracellular matrix metalloproteinase inducer
**ENZA**	Enzalutamide
**ETV4**	E26 transformation specific (ETS) variant transcription factor 4
**FAK**	Focal adhesion kinase
**GCNT3**	Glucosaminyl (N-acetyl) transferase 3
**GSK3β**	Glycogen synthase kinase 3β
**HIF**	Hypoxia-inducible factor
**HMGB1**	High mobility group box-1
**Ig**	Immunoglobulin
**IL6**	Interleukin-6
**IRAK1**	Interleukin 1 receptor associated kinase 1
**IkB**	Inhibitor kB
**JAK**	Janus kinase
**LRR**	leucine-rich region
**MAPK**	Mitogen-activated protein kinases
**MCAM**	Melanoma cell adhesion molecule
**MDSCs**	Myeloid-derived suppressor cells
**MMP**	Matrix metalloprotease
**MRP8/14**	Myeloid related protein 8/14
**mTOR**	Mammalian target of rapamycin
**NADPH**	Nicotinamide adenine dinucleotide 3-phosphate
**NPTN**	Neuroplastin
**Nrf2**	Nuclear factor E2-related factor 2
**PD-1**	Programmed death 1
**PD-L1**	PD-1 ligands
**PDZ**	Postsynaptic density-95/Discs large/Zonula occludens 1
**PI3K**	Phosphoinositide 3-kinase
**PKC**	Protein kinase C
**RAGEs**	Receptor for advanced glycation end products
**RAS**	Rat sarcoma
**ROS**	Reactive oxygen species
**SLC22A18AS**	Solute carrier family 22 member 18 antisense
**SPDEF**	SAM pointed-domain containing E26 transformation-specific transcription factor
**SSSRs**	S100 soil sensor receptors
**STAT3**	Signal transducer and activator of transcription 3
**TIR**	Toll/interleukin-1 receptor
**TLR4**	Toll-like receptor 4
**TLRs**	Toll-like receptors
**TNF-α**	Tumor necrosis factor-α
**TPL2**	Tumor progression locus 2
**TRAF2**	Tumor necrosis factor receptor-associated factor 2
**VEGFR**	Vascular endothelial growth factor receptor
**YAP**	Yes kinase-associated protein
**ZEB1**	Zinc finger E-box binding homeobox 1
